# Role of Metformin in Preventing New-Onset Chronic Kidney Disease in Patients with Type 2 Diabetes Mellitus

**DOI:** 10.3390/ph18010095

**Published:** 2025-01-14

**Authors:** Yu-Ling Lin, Sheng-Hsiang Lin, Hsi-Hao Wang, Wan-Chia Hsu, Shih-Yuan Hung, Yuan-Yow Chiou, Hung-Hsiang Liou, Min-Yu Chang, Li-Chun Ho, Ching-Fang Wu, Yi-Che Lee

**Affiliations:** 1 Division of Nephrology, Department of Internal Medicine, Hsinchu Cathay General Hospital, Hsinchu 300, Taiwan; cgh07022@cgh.org.tw; 2 Division of Nephrology, Department of Internal Medicine, Cathay General Hospital, Taipei 106, Taiwan; 3 Institute of Clinical Medicine, College of Medicine, National Cheng Kung University, Tainan 704, Taiwan; shlin922@mail.ncku.edu.tw (S.-H.L.); yuanyow@mail.ncku.edu.tw (Y.-Y.C.); 4 Department of Public Health, College of Medicine, National Cheng Kung University, Tainan 704, Taiwan; 5 Biostatistics Consulting Center, National Cheng Kung University Hospital, College of Medicine, National Cheng Kung University, Tainan 704, Taiwan; 6 Taiwan School of Medicine for International Students, College of Medicine, I-Shou University, Kaohsiung 824, Taiwan; hhgnaw@gmail.com (H.-H.W.); ed100367@edah.org.tw (S.-Y.H.); ed101366@edah.org.tw (M.-Y.C.); ed104265@edah.org.tw (L.-C.H.); ed107535@edah.org.tw (C.-F.W.); 7 Division of Nephrology, Department of Internal Medicine, E-DA Hospital, Kaohsiung 824, Taiwan; 8 Division of Endocrinology and Metabolism, Department of Internal Medicine, Kaohsiung Chang Gung Memorial Hospital, Kaohsiung 833, Taiwan; kaimu9518@cgmh.org.tw; 9 Department of Pediatrics, National Cheng Kung University Hospital, Tainan 704, Taiwan; 10 Division of Nephrology, Department of Internal Medicine, Hsin-Jen Hospital, New Taipei City 242, Taiwan; hh258527@ms23.hinet.net

**Keywords:** chronic kidney disease, diabetes mellitus, diabetic kidney disease, kidney, metformin, renal

## Abstract

**Background**: Recent evidence supports the protective role of metformin on kidney function in patients with type 2 diabetes mellitus. However, its potential to prevent new-onset chronic kidney disease (CKD) in patients with type 2 diabetes mellitus with normal renal function remains unclear. Therefore, this study aimed to investigate whether metformin could prevent the development of new-onset CKD in such patients. **Methods**: This retrospective, observational, multicenter cohort study included 316,693 patients with type 2 diabetes mellitus. After matching using the inverse probability of treatment weighting, 9109 metformin users and 1221 nonusers were analyzed. The primary outcomes were an estimated glomerular filtration rate (eGFR) of <60 mL/min/1.73 m^2^, urinary albumin-to-creatinine ratio of ≥30 mg/g, and a composite outcome defined as new-onset CKD. **Results**: The multivariable Cox survival model showed that metformin users had significantly better renal outcomes, with a notably lower risk of sustained eGFR of <60 mL/min/1.73 m^2^ (hazard ratio (HR), 0.71; 95% confidence interval (CI), 0.56–0.90) and new CKD onset (HR, 0.78; 95% CI, 0.65–0.94). **Conclusions**: Metformin plays a key role in delaying renal events in individuals with type 2 diabetes mellitus and in those with initially normal renal function.

## 1. Introduction

Metformin, an oral antidiabetic medication belonging to the biguanide class, decreases glucose absorption in the intestine, enhances glucose uptake in peripheral tissues, lowers fasting insulin levels, and improves insulin sensitivity [1]. Additionally, metformin also activates AMP-activated protein kinase (AMPK), which plays a critical role in the inhibition of gluconeogenesis [2]. Thus, AMP-activated protein kinase is essential for the regulation of energy metabolism and plays a key role in the management of diabetes and related metabolic disorders. A study highlights AMPK’s role in maintaining stable glucose levels [3]. Due to its affordability, safety profile, and cardioprotective benefits, metformin is the first-line therapy for type 2 diabetes mellitus (DM), as recommended by the American Diabetes Association and European Association for the Study of Diabetes guidelines.

Previous animal studies have shown that metformin exerts renal protection through various mechanisms [4,5,6,7,8,9,10,11,12,13,14,15,16,17]. In clinical settings, the evidence has been more variable, with some studies yielding mixed results. However, recent large-scale observational studies and meta-analyses increasingly underscore metformin’s protective role in kidney function [18,19,20,21,22,23,24,25,26]. For example, Holman et al. enrolled 5102 patients with type 2 DM and found that 10 years of intensive metformin therapy was associated with a 16% reduction in microvascular complications, including kidney damage [18]. Yi et al. examined a cohort of 1994 patients with type 2 DM and found that metformin use was consistently associated with a lower risk of overt diabetic nephropathy and major kidney events [24]. Our team recently confirmed the renoprotective effects of metformin [26]. We enrolled a large cohort of patients with type 2 DM and baseline estimated glomerular filtration rates (eGFRs) of ≥30 mL/min/1.73 m^2^ across multiple hospitals. After propensity score matching, we compared 13,096 metformin users with 13,096 nonusers, observing significantly better renal outcomes in the metformin users, including reduced risks of serum creatinine doubling, eGFR decline to ≤15 mL/min/1.73 m^2^, and progression to end-stage kidney disease (ESKD). Building on this study, we aimed to investigate whether metformin could prevent new-onset chronic kidney disease (CKD), specifically in patients with type 2 DM and normal renal function. To date, no previous studies have directly assessed the preventive effect of metformin on new CKD development in patients with initially normal renal function. By focusing on this unique population, we seek to address an important gap in the literature and further elucidate metformin’s role in protecting renal function in the early stages of diabetes.

## 2. Results

### 2.1. Baseline Characteristics

Table 1 shows the demographic characteristics of patients in the metformin and non-metformin groups, with 9109 and 1221 patients in each group, respectively. Median eGFR was 101.3 mL/min/1.73 m^2^ (84.6–120.0 mL/min/1.73 m^2^) in the metformin group and 100.8 mL/min/1.73 m^2^ (84.25–120.0 mL/min/1.73 m^2^) in the non-metformin group. Median age was 57.5 years (49.8–64.9 years) in the metformin group compared with 58.9 years (48.8–67.7 years) in the non-metformin group. Table 1 presents all the medications used for diabetes treatment at the start of the study in both groups.

### 2.2. Incidence of eGFR Persistently Below 60 mL/min/1.73 m^2^

**Figure 1:**
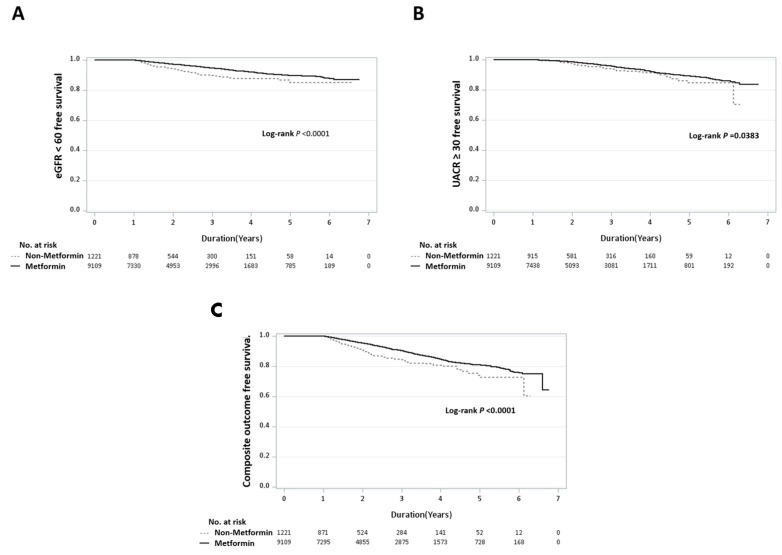
Kaplan–Meier curves for (**A**) eGFR < 60 mL/min/1.73 m^2^, (**B**) UACR ≥ 30 mg/g, and (**C**) composite outcome. After multivariable Cox proportional hazards regression analysis of variables, renoprotection effect was observed in the metformin group.

As presented in Table 2, after performing a multivariable Cox proportional hazards regression analysis, the metformin group showed a significantly lower incidence of eGFR decline to below 60 mL/min/1.73 m^2^ (HR, 0.70; 95% CI, 0.51–0.95; adjusted model 3). Additionally, a competing risk analysis was conducted using adjusted model 4, incorporating a sub-distribution hazard function to account for death as a competing risk. Time- and dose-dependent analyses were further performed using adjusted model 5, in which metformin utilization was treated as a time- and dose-dependent variable within the Cox models. These analyses indicated no significant differences in outcomes. Kaplan–Meier analysis also demonstrated that the metformin group had a lower incidence of eGFR decline to below 60 mL/min/1.73 m^2^ (*p* < 0.0001) (Figure 1A).

### 2.3. Incidence of Sustained UACR Levels of ≥30 mg/g

Table 2 summarizes the outcomes of the multivariable Cox proportional hazards regression analysis. The analysis revealed no significant difference in the occurrence of sustained UACR levels of ≥30 mg/g between the metformin and non-metformin groups throughout the follow-up period (HR, 1.00; 95% CI, 0.67–1.51; adjusted model 3). Similar results were observed in the competing risk analysis (adjusted model 4) and the time- and dose-dependent analyses (adjusted model 5). The Kaplan–Meier analysis further indicated that the metformin group had a significantly lower incidence of eGFR (≤15 mL/min/1.73 m^2^) (*p* < 0.038) (Figure 1B).

### 2.4. Incidence of New-Onset CKD

In Table 2, results from the multivariable Cox proportional hazards regression analysis showed no significant difference in the risk of developing new-onset CKD between the metformin and non-metformin groups during the follow-up period (HR, 0.90; 95% CI, 0.69–1.16; adjusted model 3). This finding was consistent when analyzed using a competing risk model (adjusted model 4). However, in the time- and dose-dependent analyses, the metformin group demonstrated a notably lower incidence of new-onset CKD (HR, 0.78; 95% CI, 0.65–0.94; adjusted model 5). Furthermore, the Kaplan–Meier analysis indicated a significantly lower incidence of ESKD in the metformin group (*p* < 0.0001) (Figure 1C).

## 3. Discussion

In this study, a cohort of 316,693 individuals with type 2 DM and normal renal function were examined to determine whether metformin confers protective benefits against new renal events. The analysis focused on three main endpoints: the incidence of persistently reduced kidney function (eGFR < 60 mL/min/1.73 m^2^), sustained UACR levels of ≥30 mg/g, and a composite outcome representing new-onset CKD. The findings revealed that metformin users had notably better renal outcomes, with a significantly lower risk of sustained eGFR decline and new CKD development. These findings indicate that metformin significantly delays renal events in individuals with type 2 DM and initially normal renal function.

Previous studies have investigated the renoprotective effects of metformin; however, the findings remain controversial [18,19,20,21,22,23,24,25]. Some studies have reported limited renal benefits. For example, De Jager et al. conducted a study among 390 patients with type 2 DM on insulin therapy supplemented with either metformin or placebo and found no significant advantage of metformin in reducing albuminuria [22]. Similarly, the ADOPT trial, led by Lachin et al., studied 4351 patients with type 2 DM treated with metformin, rosiglitazone, or glyburide, and reported no evidence of microvascular protection with metformin [23]. In contrast, other studies have reported a protective effect of metformin on kidney health. Holman et al. enrolled 5102 patients with type DM and found that 10 years of intensive metformin therapy was associated with a 16% reduction in microvascular complications, including kidney damage [18]. Amador-Licona et al. analyzed 51 patients with type 2 DM who switched from glibenclamide to metformin and observed a significant reduction in albuminuria, with an average decrease of 24.2 mg/day [20]. Yi et al. examined a cohort of 1994 patients with type 2 DM and found that metformin use was consistently associated with a lower risk of overt diabetic nephropathy and major kidney events [24]. Kwon et al. studied 10,426 patients with type 2 DM and diabetic kidney disease over a median follow-up period of 7.3 years and found that metformin use significantly reduced the risk of ESKD [21]. Recently, our group assessed the renoprotective effects of metformin in a large cohort of patients with type 2 DM and with a baseline eGFR ≥ 30 mL/min/1.73 m^2^ across multiple hospitals. After propensity score matching, 13,096 metformin users were compared with 13,096 nonusers. Our findings showed that metformin use was associated with significantly better renal outcomes, including a reduced risk of serum creatinine doubling, a decline in eGFR to ≤15 mL/min/1.73 m^2^, and a decline in progression to ESKD, with an overall hazard ratio of 0.55 for ESKD [26]. In addition, a recent systematic review and meta-analysis by the Cochrane Collaboration provided further insights into the role of metformin in preventing kidney function decline. This review included 11 randomized controlled trials (RCTs) involving 8449 participants, focusing on patients with CKD or diabetes. The results indicated that metformin may result in a slightly smaller decline in kidney function compared with placebo (mean difference 1.92 mL/min, 95% CI 0.33 to 3.51) [25]. In light of the above, accumulating evidence suggests that metformin may indeed have a protective effect on kidney function. Building on our previous study, our team aimed to further investigate metformin’s renoprotective effects. We included patients with type 2 DM who had completely normal kidney function at baseline and focused on whether metformin could prevent new-onset CKD. This approach provides a novel perspective, as no previous studies have specifically examined the preventive effects of metformin on new-onset CKD in patients with initially normal renal function.

It is particularly noteworthy that to ensure the safety and efficacy of metformin in CKD prevention, its use requires adjustment and close monitoring based on the patient’s eGFR to reduce the risk of lactic acidosis. According to the consensus report by the American Diabetes Association (ADA) and the Kidney Disease: Improving Global Outcomes (KDIGO) organization [27,28], patients with CKD should have their eGFR monitored at least once a year. If eGFR falls below 60 mL/min/1.73 m^2^, it is recommended to increase the monitoring frequency to every 3 to 6 months. For patients with an eGFR between 30 and 44 mL/min/1.73 m^2^, the metformin dose should be reduced to 1000 mg per day. Additionally, for patients with an eGFR between 45 and 59 mL/min/1.73 m^2^ who have comorbidities that increase the risk of lactic acidosis (such as hypoperfusion or hypoxemia), a dose reduction should also be considered. Furthermore, most cases of metformin-associated lactic acidosis occur when patients experience acute illness, often due to acute kidney injury (AKI) leading to reduced metformin clearance.

This highlights the importance of balancing the potential renoprotective benefits of metformin with the need for vigilant monitoring, particularly in patients at risk of renal function decline.

The mechanism underlying the potential renoprotective effects of metformin remains incompletely understood, likely involving multiple factors. Several possible explanations for metformin’s protective role in DKD have been proposed [5,6,7,10,14,15,29,30,31,32,33,34,35]. First, research indicates that metformin may help reduce glomerulosclerosis and fibrosis. In studies involving diabetic rats, metformin has been shown to inhibit kidney transforming growth factor-beta expression and extracellular matrix production by reducing oxidative stress and inflammation, which in turn lowers proteinuria [6]. Metformin also reduces high-glucose-induced NF-κB activation in vitro [31]. In a DKD rat model, metformin exhibited renoprotective effects by reducing tubular injury and collagen accumulation [29]. Its AMP-activated protein kinase-dependent renal protection has been validated in a CKD model [5]. Secondly, studies suggest that metformin may reduce tubular injury [10,32,33], as it attenuates apoptosis in renal tubular cells by inhibiting NF-κB activation and reducing ROS production [34,35]. Additionally, Kim et al. demonstrated that metformin prevents lipotoxicity-induced apoptosis mediated by glucagon-like peptide-1 receptor downregulation both in vitro and in vivo [7]. Third, previous studies suggested that metformin enhances autophagy and reduces renal histological changes by activating the AMPK/Sirt1/FoxO1 signaling pathway [14,15]. Furthermore, a study has shown that metformin effectively slows non-diabetic CKD progression in rats, halts functional decline, and reduces inflammation. This kidney protection is linked to activation of the Hippo signaling pathway, indicating the potential of metformin in CKD treatment [30]. Collectively, these findings suggest that the renoprotective effects of metformin are mediated by promoting autophagy and reducing inflammation, oxidative stress, and fibrosis.

This study has certain limitations. As this was an observational cohort study rather than a randomized controlled trial, randomized methods are inherently lacking. Although propensity score matching was used to balance the differences between the metformin and non-metformin groups, unmeasured confounding factors may have influenced the results. Additionally, the diagnosis of ESKD and other comorbidities relied on administrative claims data, which introduced the potential for misclassification.

This extensive multicenter retrospective cohort study demonstrated that metformin effectively prevented the onset of new-onset CKD in patients with type 2 DM and normal kidney function.

## 4. Materials and Methods

### 4.1. Study Design

This was a multicenter, retrospective, observational cohort study. Patients who received diabetes treatment at the institution during a defined timeframe were categorized based on their use of metformin. Changes in kidney function over time were tracked and compared between groups.

### 4.2. Database and Study Sample

**Figure 2:**
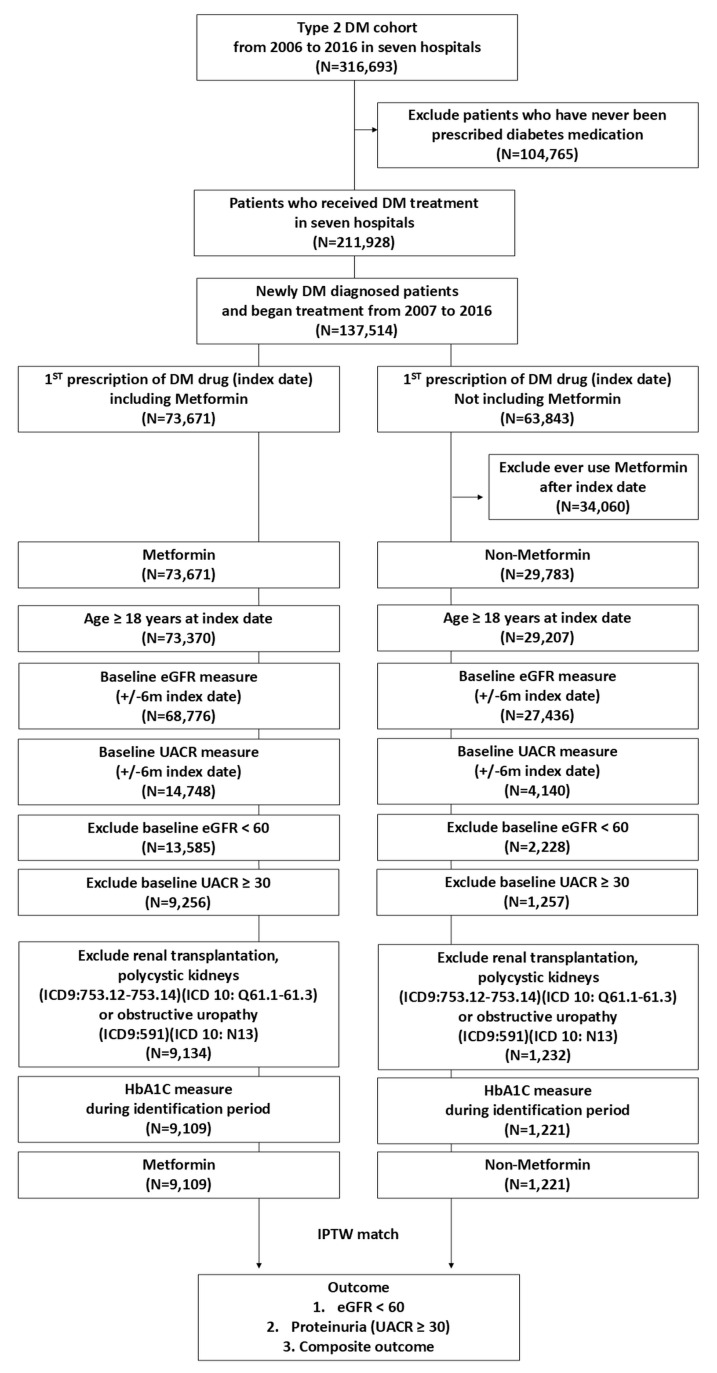
Study flow chart of metformin effect on kidney function.

This retrospective cohort study included patients with type 2 DM treated at the Chang Gung Memorial Hospital, a network of seven medical institutions across Northeastern and Southern Taiwan. The study period spanned between January 2006 and December 2016, encompassing 316,693 patients. De-identified data, including patient demographics (birth date and sex), diagnostic codes based on ICD-9 and ICD-10-CM, medication prescriptions, and laboratory test results, were extracted from electronic medical records. Patients with type 2 DM between January 2006 and December 2016 were included in the analysis (n = 316,693) (Figure 2). Patients who did not receive any diabetes medication during the identification period (n = 104,765) were excluded (deemed to refuse medicine control, not requiring drug treatment, or receiving treatment in an external hospital). Subsequently, only patients who were newly diagnosed with type 2 DM and who began treatment between January 2007 and December 2016 were included in the study (n = 137,514). Patients undergoing treatment for diabetes before January 2007 were excluded from the study. The patients were then classified into two groups based on whether metformin was included in their initial diabetes prescription. Patients in a non-metformin group who received metformin later were excluded from the analysis. Further exclusion criteria were patients aged < 18 years and those lacking baseline eGFR and urine albumin-to-creatinine ratio (UACR) data. Patients with a baseline eGFR of <60 mL/min/1.73 m^2^ and those with a UACR of ≥30 mg/g were then excluded. Additionally, patients with a history of renal transplantation, polycystic kidney disease, or obstructive uropathy, as well as those with missing glycated hemoglobin (HbA1C) data within the identification period, were excluded. Finally, metformin (n = 9109) and non-metformin (n = 1221) groups were enrolled. The final step involved matching the two groups of patients using an inverse probability of treatment weighting (IPTW) approach that accounted for baseline eGFR category, UACR, age, sex, DM duration, comorbidities, and initial medication.

### 4.3. Matching

In this study, we utilized IPTW to enhance the comparability between the groups. Matching criteria included baseline eGFR category, UACR, age, sex, duration of DM, comorbidities (such as hypertension, hyperlipidemia, coronary artery disease, cerebrovascular disease, congestive heart failure, gout, nephrolithiasis, chronic hepatitis and liver cirrhosis, autoimmune disorders, chronic lung disease, and malignancy), and initial medications (metformin, sulfonylurea, meglitinide, alpha-glucosidase inhibitors (AGIs), thiazolidinediones (TZDs), dipeptidyl peptidase-4 (DPP4) inhibitors, insulin, statins, anti-gout drugs, calcium channel blockers (CCBs), diuretics, aspirin, and non-steroidal anti-inflammatory drugs (NSAIDs)) [36,37,38,39,40,41,42,43,44,45]. The IPTW did not include adjustments for glucagon-like peptide-1 (GLP1) receptor agonists or sodium-glucose cotransporter type 2 (SGLT2) inhibitors because of the low proportion of patients who initially used these medications, as shown in the index data.

### 4.4. Data Collection

The eGFR was derived from serum creatinine using the CKD epidemiological collaboration equation [46].

### 4.5. Outcome

Primary outcomes were defined as an eGFR persistently <60 mL/min/1.73 m^2^ for at least three consecutive months, a UACR level consistently ≥30 mg/g for the same period, or the occurrence of either condition for three consecutive months, regardless of the order in which they appeared.

### 4.6. Duration of Follow-Up/Censor Date

The follow-up periods and censoring dates differed among the participant groups in this study. For individuals who maintained continuous medical care until the close of the 2016 database, follow-up continued until the end date of the database, which marked the censoring point. Participants who reached an outcome prior to the conclusion of the database were followed up until the event occurred. Those who died during the follow-up period were monitored until the date of death, and individuals lost to follow-up were tracked until the date of their last recorded medical visit.

### 4.7. Statistical Analysis

To compare baseline characteristics, we used the χ^2^ test for categorical variables and unpaired Student’s *t*-test for continuous variables. Hazard ratios (HRs) and 95% confidence intervals (CIs) were estimated using the Cox proportional hazards model, and multivariable Cox proportional hazards models were used to assess the effect of metformin on renal outcomes. Follow-up time was calculated from therapy initiation to death or the last recorded follow-up. The study employed a hierarchical analysis sequentially incorporating comorbidities, laboratory data, and medication use. The selection of parameters for comorbidities and medication use was based on the absolute standardized mean difference (aSMD), as shown in Table 1. An aSMD > 0.1 indicated that the distribution of this factor between the two groups was imbalanced, necessitating its inclusion in the model for adjustment. Laboratory data were treated as continuous variables. Even if no significant difference was observed between groups in Table 1, residual confounding could still exist. Therefore, all laboratory data were included as continuous variables in the model. Five models were developed for the analysis. Model 1 was adjusted for age, sex, and comorbidities with an absolute standardized mean difference > 0.1. Model 2 included adjustments for age, sex, comorbidities, and laboratory values, such as systolic and diastolic blood pressure, total cholesterol, low- and high-density lipoprotein cholesterols, triglycerides, hemoglobin, and HbA1c. Model 3 expanded on these by adding medications used throughout the follow-up, including sulfonylureas, meglitinides, AGIs, TZDs, DPP4 inhibitors, GLP1 agonists, SGLT2 inhibitors, insulin, angiotensin-converting enzyme inhibitors, angiotensin-receptor blockers, renin inhibitors, statins, anti-gout drugs, CCBs, beta blockers, diuretics, aspirin, and NSAIDs. A competing risk analysis was conducted using model 4, incorporating a sub-distribution hazard function to account for death as a competing risk. Model 5 utilized a Cox model that incorporated time-varying covariates to account for both time and dose dependencies. Kaplan–Meier survival curves were constructed to assess time-to-event outcomes, and differences between groups were evaluated using the Log-rank test. All statistical analyses were performed using SAS software (version 9.4; SAS Institute Inc., Cary, NC, USA) and R version 3.0.3 (R Foundation for Statistical Computing).

## Figures and Tables

**Table 1:** Demographic characteristics of metformin and non-metformin cohorts before and after matching.

Variables	Before Matching			After Matching		
	Metformin (N = 9109)	Non-Metformin (N = 1221)	aSMD	Metformin	Non-Metformin	aSMD
	n (%)/Median (IQR)	n (%)/Median (IQR)		n (%)/Median (IQR)	n (%)/Median (IQR)	
Baseline eGFR, mL/min/1.73 m^2^			0.157			0.032
60–<70	633 (6.95)	135 (11.06)		670 (7.30)	61 (7.33)	
70–<80	1057 (11.60)	147 (12.04)		1062 (11.58)	91 (10.90)	
80–<90	1321 (14.50)	149 (12.20)		1306 (14.24)	120 (14.43)	
≥90	6098 (66.94)	790 (64.70)		6133 (66.88)	560 (67.34)	
Median (IQR)	100.97 [84.81,120.00]	100.74 [81.33,120.00]	0.036	101.30 [84.68,120.00]	100.85 [84.25,120.00]	0.001
Baseline UACR, mg/dL	9.20 [6.00,15.00]	9.90 [6.20,16.70]	0.106	9.30 [6.00,15.20]	9.80 [6.30,16.40]	0.077
Age			0.203			0.070
<45	1407 (15.45)	233 (19.08)		1437 (15.67)	120 (14.42)	
45–<55	2323 (25.50)	251 (20.56)		2297 (25.05)	200 (24.08)	
55–<65	3126 (34.32)	357 (29.24)		3102 (33.83)	289 (34.80)	
≥65	2253 (24.73)	380 (31.12)		2334 (25.45)	222 (26.71)	
Median (IQR)	57.56 [49.81,64.90]	58.98 [48.81,67.75]	0.038	57.67 [49.79,65.13]	58.68 [50.36,65.93]	0.086
DM duration (months)	0.23 [0.00,5.88]	0.62 [0.00,11.96]	0.114	0.23 [0.00,6.01]	0.49 [0.00,9.92]	0.038
Follow-up duration (years)	2.35 [1.25,3.64]	1.96 [1.03,3.16]	0.228	2.32 [1.23,3.60]	1.95 [1.00,3.21]	0.231
Male	5099 (55.98)	685 (56.10)	0.002	5158 (56.25)	481 (57.89)	0.033
Comorbidity						
Hypertension	3196 (35.09)	399 (32.68)	0.051	3215 (35.06)	287 (34.55)	0.011
Hyperlipidemia	2869 (31.50)	322 (26.37)	0.113	2846 (31.04)	243 (29.23)	0.039
Coronary artery disease	757 (8.31)	93 (7.62)	0.026	772 (8.42)	73 (8.73)	0.011
Cerebrovascular disease	547 (6.01)	104 (8.52)	0.097	586 (6.39)	59 (7.09)	0.028
Congestive heart failure	196 (2.15)	33 (2.70)	0.036	204 (2.22)	20 (2.40)	0.012
Gout	366 (4.02)	44 (3.60)	0.022	370 (4.03)	32 (3.90)	0.007
Nephrolithiasis	147 (1.61)	12 (0.98)	0.056	139 (1.52)	11 (1.36)	0.014
Chronic hepatitis and liver cirrhosis	1406 (15.44)	195 (15.97)	0.015	1405 (15.33)	117 (14.08)	0.035
Autoimmune disorders	76 (0.83)	15 (1.23)	0.039	80 (0.87)	7 (0.86)	0.001
Chronic lung disease	462 (5.07)	57 (4.67)	0.019	448 (4.89)	35 (4.26)	0.030
Malignant	1109 (12.17)	161 (13.19)	0.030	1118 (12.19)	97 (11.64)	0.017
Laboratory data						
SBP, ≥140 mmHg	3691 (41.01)	409 (34.08)	0.143	3690 (40.73)	317 (38.71)	0.041
DBP, ≥90 mmHg	1389 (15.43)	117 (9.75)	0.172	1365 (15.06)	88 (10.70)	0.131
TC, ≥200 mg/dL	3287 (36.51)	335 (27.99)	0.183	3250 (35.87)	235 (28.96)	0.148
LDL, ≥100 mg/dL	5784 (64.19)	629 (52.68)	0.235	5770 (63.60)	440 (54.27)	0.190
HDL, ≥40 mg/dL	6257 (69.92)	809 (68.27)	0.036	6227 (69.11)	555 (68.81)	0.007
TG, ≥150 mg/Dl	3520 (39.14)	348 (29.22)	0.210	3541 (39.11)	267 (32.89)	0.130
Hb, ≥12 g/dL	6589 (86.99)	751 (74.88)	0.312	6585 (86.15)	545 (80.24)	0.159
HbA1C			0.150			0.100
≥9%	2562 (28.13)	396 (32.43)		2730 (29.77)	268 (32.26)	
8%–<9%	1228 (13.48)	198 (16.22)		1248 (13.61)	133 (16.02)	
7%–<8%	2673 (29.34)	296 (24.24)		2631 (28.69)	209 (25.14)	
<7%	2646 (29.05)	331 (27.11)		2561 (27.93)	221 (26.59)	
Initial medications in the index data						
Sulfonylurea, yes	1215 (13.34)	259 (21.21)	0.209	1395 (15.21)	274 (32.95)	0.424
Meglitinide, yes	66 (0.72)	80 (6.55)	0.315	146 (1.59)	20 (2.42)	0.059
AGIs, yes	181 (1.99)	187 (15.32)	0.488	381 (4.16)	53 (6.40)	0.100
TZD, yes	144 (1.58)	46 (3.77)	0.136	195 (2.13)	48 (5.82)	0.190
DPP4 inhibitor, yes	1906 (20.92)	456 (37.35)	0.367	2263 (24.68)	391 (47.05)	0.480
GLP1, yes	2 (0.02)	3 (0.25)	0.061	3 (0.03)	4 (0.48)	0.088
SGLT2 inhibitor, yes	1 (0.01)	9 (0.74)	0.119	1 (0.01)	24 (2.91)	0.243
Insulin, yes	126 (1.38)	338 (27.68)	0.804	401 (4.37)	58 (6.96)	0.112
ACEI, yes	491 (5.39)	49 (4.01)	0.065	487 (5.31)	41 (4.95)	0.016
ARB, yes	3170 (34.80)	425 (34.81)	0	3209 (35.00)	310 (37.34)	0.049
Renin inhibitor, yes	30 (0.33)	4 (0.33)	0	31 (0.34)	2 (0.22)	0.023
Statin, yes	3085 (33.87)	322 (26.37)	0.164	3051 (33.27)	270 (32.46)	0.017
Anti-gout, yes	264 (2.90)	38 (3.11)	0.013	265 (2.88)	22 (2.68)	0.012
CC blockers, yes	1658 (18.20)	191 (15.64)	0.068	1644 (17.93)	141 (16.95)	0.026
Beta blockers, yes	1608 (17.65)	206 (16.87)	0.021	1601 (17.46)	137 (16.54)	0.024
Diuretics, yes	1115 (12.24)	162 (13.27)	−0.031	1127 (12.29)	95 (11.43)	0.026
Aspirin, yes	1420 (15.59)	205 (16.79)	0.033	1481 (16.15)	159 (19.10)	0.078
NSAID, yes	800 (8.78)	85 (6.96)	0.068	783 (8.54)	59 (7.10)	0.053

IPTW (stabilized IPTW-ATE weights) include eGFR (baseline category), UACR, age, gender, DM duration, comorbidity and initial medication. aSMD denotes absolute standardized mean difference, eGFR estimated glomerular filtration rate, SBP systolic blood pressure, DBP diastolic blood pressure, TC total cholesterol, LDL low-density lipoprotein, HDL high-density lipoprotein, TG triglyceride, Hb hemoglobin, HbA1C glycated hemoglobin, UA uric acid, AGIs alpha-glucosidase inhibitors, TZD thiazolidinediones, DPP4 dipeptidyl peptidase-4, GLP1 glucagon-like peptide-1, SGLT2 sodium-glucose cotransporter type 2, ACEI angiotensin-converting enzyme inhibitor, ARB angiotensin-receptor blocker, CCB calcium channel blockers, and NSAID non-steroidal anti-inflammatory drug.

**Table 2:** Adjusted hazard ratios (HRs) of renal outcome among the cohort of sampled patients during the follow-up years.

Type of Treatment (No. of Patients)	No. of Events	Incidence Rate per 100 Patient-Years	Study Outcome, HR (95% CI)
eGFR < 60 mL/min/1.73 m^2^			
Nonuser (n = 1221)	72	2.89	
Metformin user (n = 9109)	383	1.72	
Model 1 ^a^			0.68 (0.50–0.91)
Model 2 ^b^			0.64 (0.47–0.86)
Model 3 ^c^			0.70 (0.51–0.95)
Model 4 ^d^			0.71 (0.51–0.98)
Model 5 ^e^			0.71 (0.56–0.90)
Proteinuria (UACR ≥ 30)			
Nonuser (n = 1221)	50	1.93	
Metformin user (n = 9109)	355	1.56	
Model 1 ^a^			0.82 (0.57–1.17)
Model 2 ^b^			0.95 (0.64–1.42)
Model 3 ^c^			1.00 (0.67–1.51)
Model 4 ^d^			1.03 (0.69–1.55)
Model 5 ^e^			0.77 (0.59–1.01)
Composite outcome			
Nonuser (n = 1221)	117	4.82	
Metformin user (n = 9109)	716	3.27	
Model 1 ^a^			0.76 (0.60–0.97)
Model 2 ^b^			0.82 (0.64–1.05)
Model 3 ^c^			0.90 (0.69–1.16)
Model 4 ^d^			0.91 (0.69–1.20)
Model 5 ^e^			0.78 (0.65–0.94)

^a^ Adjusted for age, sex, and comorbidity (aSMD ≥ 0.1). ^b^ Adjusted for age, sex, comorbidity (aSMD ≥ 0.1), and laboratory data. ^c^ Adjusted for age, sex, comorbidity (aSMD ≥ 0.1), laboratory data, and medication (aSMD ≥ 0.1) ever used throughout the entire follow-up period. ^d^ Sub-distribution hazard function adjusted for age, sex, comorbidity (aSMD ≥ 0.1), laboratory data, and medication (aSMD ≥ 0.1). ^e^ Time-dependent Cox mode.

## Data Availability

The data that support the findings of this study are available from Kaohsiung Chang Gung Memorial Hospital, but restrictions may apply to the availability of these data, which were approved by the individual hospital IRB for the current study and thus are not publicly available. However, processed datasets can be requested and made available by the authors with the permission of the Kaohsiung Chang Gung Memorial Hospital.
